# Evaluating the impact of multilevel evidence-based implementation strategies to enhance provider recommendation on human papillomavirus vaccination rates among an empaneled primary care patient population: a study protocol for a stepped-wedge cluster randomized trial

**DOI:** 10.1186/s13012-018-0778-x

**Published:** 2018-07-13

**Authors:** Lila J. Finney Rutten, Carmen Radecki Breitkopf, Jennifer L. St. Sauver, Ivana T. Croghan, Debra J. Jacobson, Patrick M. Wilson, Jeph Herrin, Robert M. Jacobson

**Affiliations:** 1 Division of Health Care Policy and Research, Mayo Clinic, Rochester, MN USA; 2 Robert D. and Patricia E. Kern Center for Science of Health Care Delivery, Mayo Clinic, Rochester, MN USA; 3 Division of Epidemiology, Mayo Clinic, Rochester, MN USA; 4 Nicotine Dependence Center, Mayo Clinic, Rochester, MN USA; 5 Division of Biomedical Statistics and Informatics, Mayo Clinic, Rochester, MN USA; 6 Yale University, New Haven, CT USA; 7 Department of Pediatric and Adolescent Medicine, Mayo Clinic, Rochester, MN USA

**Keywords:** Adolescent, Child, Feedback, Human papillomavirus vaccines, Immunization, Medical audit, Parents, Primary health care, Reminder systems, Vaccines

## Abstract

**Background:**

Each year, human papillomavirus (HPV) causes 30,000 cancers in the USA despite the availability of effective and safe vaccines. Uptake of HPV vaccine has been low and lags behind other adolescent vaccines. This protocol describes a multilevel intervention to improve HPV vaccination rates.

**Methods:**

Using a cluster randomized trial, we will evaluate the independent and combined impact of two evidence-based implementation strategies with innovative enhancements on HPV vaccination rates for female and male patients. The clusters are six primary care sites providing care to pediatric populations. We will use a stepped-wedge cluster randomized design, including process evaluation, to test the hypothesis that compared with the current course of care and a practice-level intervention using reminder-recall interventions coupled with provider-level audit and feedback with education increases HPV vaccination rates in exposed clusters. The factorial design allows us to use a single trial to test these two interventions and to assess each individually and in combination. Our design has four 12-month steps. The first step will be a baseline period; data collected during it will provide a within-practice control group for each cluster. Second, two clusters will be randomly assigned to receive intervention 1 (reminder and recall), and two clusters will be randomly selected to receive intervention 2 (audit and feedback with education). Third, the other two clusters will be randomly allocated to intervention 1 or 2. Clusters initially with intervention 1 will be randomly allocated to 1 + 2 or 1; clusters initially with intervention 2 will be randomly allocated to 1 + 2 or 2. Fourth, all clusters will receive both interventions. To ensure balance of patient numbers across interventions, we will use block randomization at the first step, with the six clusters grouped into three pairs according to volume. Our primary outcome will be vaccination rates.

**Discussion:**

Results of our clinical trial and process evaluation will provide evidence showing whether practice- and provider-level interventions improve HPV vaccination rates and will offer insight into contextual factors associated with direction and magnitude of trial outcomes.

**Trial registration:**

ClinicalTrials.gov, NCT03501992, registered April 18, 2018.

**Electronic supplementary material:**

The online version of this article (10.1186/s13012-018-0778-x) contains supplementary material, which is available to authorized users.

## Background

From 2008 through 2012, on average, 38,793 new cases of human papillomavirus (HPV)-associated cancers occurred each year in the USA [1]. This represents an increase in the number of cases from 2004 through 2008, when the number calculated was 33,369 new cases [2]. The observed increase in HPV-associated cancers has occurred despite the recommendation for universal vaccination against HPV [3, 4].

Recommendations call for universal vaccination of children 11 to 12 years of age with the two-dose series (or three-dose series if immunocompromised) over 6 to 12 months, with universal catch-up for girls and women age 13 through 26 years and boys and men age 13 through 21 years [5]. However, nationally, HPV vaccine delivery rates continue to be low, have lagged in contrast to other vaccines for adolescents [6], and fail to approach Healthy People 2020 targets, which are 80% for girls and boys age 13 to 15 years [7]. HPV vaccination rates in our local population are similarly low [8]. Efforts to improve vaccination rates are urgently needed [9].

### Study objectives

We propose to address this critical need through development, implementation, and evaluation of a multilevel intervention, leveraging evidence-based implementation strategies to increase HPV vaccination rates in an empaneled primary care patient population. Multilevel interventions offer not just the combination of incremental effects of individual interventions adopted simultaneously but also synergistic effects [10, 11]. Using a stepped-wedge cluster randomized trial, we will assess the independent and synergistic effect of a multilevel intervention on HPV vaccination rates. We also will conduct a process evaluation to assess the fidelity and quality of intervention implementation and evaluate potential factors that may be associated with observed outcomes.

#### Multilevel intervention framework

The Social Ecological Model (SEM) is a theoretical framework for understanding multiple and interactive personal and environmental factors that influence behavior [12–14]. Applying SEM to the clinical setting, we will implement two evidence-based interventions, each with novel components, to influence organizational, interpersonal, and individual levels of behavior.

#### Obstacles to HPV vaccination and strategies for improvement

A rich body of research has identified obstacles at the organizational, interpersonal, and individual levels that have impeded the USA from achieving national goals with HPV vaccination [15, 16].

##### Organizational barriers

Clinical visits among adolescents are infrequent. Yet, even when such visits occur, patients eligible for a dose of HPV vaccine often do not receive it. These missed opportunities occur without regard to race/ethnicity and spoken language in the USA [17, 18].

##### Organizational interventions

Reminders that the vaccination is due now or soon and recalls indicating a patient is late for a vaccination dose have been shown repeatedly to be effective in improving vaccination rates [19–28]. Reminders and recalls overcome major barriers created by the lack of adolescent health care visits [29] and of patient and parent awareness of the vaccine by offering the provider’s recommendation and providing the date when the next dose is due [30, 31]. Our reminder-recall communication will address message content and tone, adopting expectant (announcement-style or presumptive) language to make a strong recommendation for the vaccine, and it will describe benefits of early vaccination. Both these approaches have been shown to be effective in communication around vaccination [32–38].

Standing orders or nurse protocols also have proven effective for increasing vaccination rates [19]. These orders permit nurses to vaccinate patients without a provider’s order and support nurse visits without clinical examinations. Thus, standing orders improve access to vaccination and simplify the vaccination process. However, in our system, parents are not systematically informed of the availability and convenience of nurse visits for vaccination. Therefore, we will include this information in the reminder-recall communication, to bring attention to the convenience of scheduling nurse visits for HPV vaccination.

Point-of-care prompts can take the form of a nurse’s review of the current vaccination record at every patient visit and can flag recommended or past-due vaccines in the health record for the provider to address at that visit. Point-of-care prompts have been demonstrated to be effective for the HPV vaccine [21, 26, 39].

Audit and feedback interventions also have shown effectiveness for HPV vaccination [21, 26, 39–42]. These interventions involve the regularly presenting clinicians with their performance metrics. We propose to enhance this effective strategy by adopting a social pressure approach wherein providers will be given specific feedback on their performance and on the performance of their colleagues [43, 44].

##### Interpersonal barriers

The presence and quality of provider recommendation for HPV vaccination affect vaccination rates; provider recommendations have been shown across the spectrum of vaccines to result in increased vaccination rates [30, 45–54]. Furthermore, investigation has shown not only that the presence of the recommendation matters but also that the strength of the recommendation matters [52].

##### Interpersonal interventions

The Advisory Committee on Immunization Practices (ACIP) has identified the need for providers to strengthen their recommendations for HPV vaccinations [55, 56]. At the interpersonal level, our intervention includes a tool kit for providers that illustrates techniques for making a strong recommendation for HPV vaccination and for addressing questions commonly asked by patients and parents.

##### Individual barriers

An important individual-level barrier to HPV vaccination is pain, often expressed as *a fear of needles* or not wanting to receive a shot [49]. Injection-site pain is the most common adverse effect reported by recipients of the HPV vaccine [57]. Walter et al. [58] found modest increases in pain reported by girls age 9 to 18 years with HPV vaccine compared with concomitantly administered vaccines. Burns et al. [59] similarly found that approximately two thirds of the time, HPV vaccination was more painful than concomitant vaccinations given to children age 10 to 18 years.

##### Individual interventions

A comprehensive systematic review has demonstrated the ability through various measures to reduce pain associated with vaccination [60]. Two systematic reviews showed substantial evidentiary support for topical anesthetics and external cold combined with vibration to reduce vaccination pain [61, 62]. Despite the evidence supporting topical anesthetics with vaccination, they are rarely used [63]. Therefore, we will routinely offer pain-free vaccine administration and ensure that parents and patients learn of this practice through the reminder-recall communication.

## Methods design

### Research ethics approval

The protocol described herein was approved as a minimal risk study by the Mayo Clinic Institutional Review Board (IRB) (No. 17-010661).

#### Protocol amendments

This protocol was registered with ClinicalTrials.gov on April 18, 2018 (No. NCT03501992). Results of the study will be reported to ClinicalTrials.gov. The study team will meet weekly to monitor the study and record study progress in the minutes of those meetings. The team will regularly review protocol compliance. The corresponding principal investigator will submit annual reports on study progress to the Mayo Clinic IRB. The principal investigator will report any unexpected and serious adverse events immediately to the IRB and will follow IRB policy and procedures regarding any unanticipated problem involving risk to participants or other persons or any protocol violations.

### Trial design

We will test the individual and synergistic effects of novel, multilevel HPV vaccine delivery implementation interventions on rates of initiation and completion of HPV vaccinations in six clinical practices, using a stepped-wedge cluster randomized trial with process evaluation [64–67]. This will enable the evaluation of both main and synergistic effects. We will also collect rich process information to inform dissemination and implementation efforts that in turn will inform interpretation of the success of the interventions.

The stepped-wedge design permits us to test the presence of each intervention in each primary care practice, allowing each practice to serve as its own control, thereby reducing bias due to imbalanced risk factors across practices. The incorporation of a factorial design allows us to use a single trial to test two interventions and assess them individually and in combination [68, 69]. This design also provides opportunity to conserve sample size while maintaining statistical power [70]. In addition, we will collect data from parents and providers about their experiences with the intervention components. We will use these process measures along with provider- and clinic-level characteristics to analyze outcomes and make adjustments necessary for future dissemination.

### Study aims

Over 5 years, we will evaluate two evidence-based implementation interventions with innovative enhancements in six primary care practices to evaluate their individual and combined effect on rates of HPV vaccination among female and male patients. Specifically, we propose to address the following aims and hypotheses.

#### Aim 1

Test the hypothesis that, compared with no intervention (current practice), a practice-level intervention using reminder-recall communication featuring the availability of nonmedication and medication anesthetics, the convenience of nurse-only visits, and the use of persuasive language for early on-time vaccinations will improve the odds of a child receiving an HPV vaccine dose by at least 20%.

#### Aim 2

Test the hypothesis that, compared with no intervention (current practice), a provider-level intervention using missed opportunities audit and feedback applying social pressure (peer-performance comparisons) and equipping providers with a strong-recommendation tool kit will improve the odds of a child receiving an HPV vaccine dose by at least 20%.

#### Aim 3

Test the hypothesis that simultaneous implementation of interventions targeting individual, interpersonal, and organizational factors will have a synergistic effect on HPV vaccine delivery rates, more than doubling the odds of a child receiving an HPV vaccine dose.

### Research setting

The study will be conducted in six primary care practices at Mayo Clinic in Rochester, Minnesota, and Kasson, Minnesota (Appendix A). These practices are part of Mayo Clinic’s Employee and Community Health primary care initiative and employ salaried pediatricians, pediatric nurse practitioners, family physicians, and family medicine nurse practitioners (Appendix B). All six practices provide care to children, including those in the trial’s target age group of 11 to 12 years. Table 1 reports the recent *incidence* rates of initiation and completion of the HPV vaccine series in the six participating practices. These reflect the numbers used in our power calculations. Table 2 reports the recent *prevalence* rates of the same. Uniformity in critical features across the six clinical practices will enhance the success and efficiency of the study and contribute to the fidelity of the intervention delivery across time, providers, and clinics, as well as the interpretability of the results.

**Table 1:** Rates of incidence of human papillomavirus vaccine Initiation and of completion in participating practices

		Initiation, no. (%)		Completion, no. (%)	
Practice	Age, years	2015	2016	2015	2016
A	11–12	257 (22.1)	299 (24.3)	179 (10.1)	227 (11.8)
	13–17	166 (13.4)	170 (14.1)	237 (10.2)	232 (10.0)
B	11–12	140 (23.6)	152 (24.8)	41 (5.9)	55 (7.2)
	13–17	148 (15.2)	156 (16.1)	77 (12.3)	126 (8.7)
C	11–12	69 (13.3)	83 (16.2)	39 (6.0)	37 (5.8)
	13–17	82 (9.4)	81 (9.4)	66 (5.4)	71 (5.7)
D	11–12	186 (23.1)	207 (24.3)	68 (6.6)	106 (9.4)
	13–17	116 (12.3)	161 (17.6)	76 (5.6)	117 (8.2)
E	11–12	158 (20.3)	197 (26.2)	70 (7.3)	93 (10.1)
	13–17	148 (13.2)	173 (17.6)	114 (7.2)	161 (11.1)
F	11–12	93 (23.3)	108 (28.3)	55 (9.9)	51 (10.0)
	13–17	73 (12.7)	83 (18.5)	57 (6.8)	77 (11.4)
Total	11–12	903 (21.2)	1046 (24.1)	559 (5.2)	569 (9.6)
	13–17	733 (12.8)	824 (15.3)	452 (8.0)	784 (9.1)

**Table 2:** Rates of prevalence of human papillomavirus vaccine initiation and of completion in participating practices

Practice	Age, years	Initiation, no. (%)		Completion, no. (%)	
		2015	2016	2015	2016
A	11–12	1326 (59.5)	1441 (60.7)	636 (28.5)	668 (28.2)
	13–17	2912 (73.2)	3121 (75.1)	1903 (47.8)	2064 (50.0)
B	11–12	309 (40.6)	383 (45.4)	105 (13.8)	131 (15.5)
	13–17	902 (52.3)	1118 (58.0)	496 (28.8)	607 (31.5)
C	11–12	277 (38.1)	287 (40.0)	115 (15.8)	119 (16.6)
	13–17	890 (53.0)	979 (55.6)	511 (30.5)	581 (33.0)
D	11–12	550 (47.1)	639 (49.7)	212 (18.2)	257 (20.0)
	13–17	1139 (58.0)	1330 (63.8)	683 (34.8)	775 (37.2)
E	11–12	442 (41.6)	448 (44.7)	169 (15.9)	173 (17.3)
	13–17	1242 (56.1)	1269 (61.1)	751 (33.9)	786 (37.8)
F	11–12	341 (52.7)	296 (51.9)	148 (22.9)	113 (19.8)
	13–17	811 (61.8)	652 (64.1)	533 (40.6)	419 (41.2)
Total	11–12	3245 (49.2)	3494 (51.4)	1385 (21.0)	1461 (21.5)
	13–17	7896 (61.3)	8469 (65.0)	4877 (37.9)	5232 (40.2)

### Eligibility

Eligibility at the practice level was determined by two criteria: primary care practice in the Mayo Clinic Employee and Community Health practice and provision of care to age-eligible children and adolescents. The Consolidated Standards of Randomized Trials flow diagram is shown in Additional file 1.

Eligible patients are children age 11 and 12 years who in the previous calendar month became eligible for one dose of the HPV vaccine. Review of the patient’s electronic health record at Mayo Clinic will determine whether the patient is due for a dose of the vaccine. The six practices currently conduct such scans for the point-of-care prompts to determine whether the patient presenting for care is due for an HPV vaccine dose. Patients eligible for this study would be age 11 or 12 years at the first day of each 12-month step. Thus, patients eligible for the study and due for the vaccination comprise three groups: those who received no valid HPV vaccine, those who received one valid HPV dose and five calendar months or more have passed since that dose, and those who received two valid doses, but the second dose was given less than 5 months after the first. A *valid dose* includes a dose administered when the patient was age 9 years or older (the minimum age per ACIP) and that meets the minimum interval [5].

### Allocation

Participating practices will be allocated as illustrated in Table 3. Our design has four 12-month steps, for a total of 48 months. To ensure that patient numbers balance across the interventions, we will group the six practices into three pairs according to volume and will block randomize these pairs.

**Table 3:** Factorial design used in the proposed stepped-wedge cluster randomized trial

Practice	Step^a^			
	1	2	3	4
A	0	0	1	1 + 2
B	0	1	1 + 2	1 + 2
C	0	2	2	1 + 2
D	0	0	2	1 + 2
E	0	1	1	1 + 2
F	0	2	1 + 2	1 + 2

^a^0, current care; 1, reminder and recall; 2, audit and feedback and strong recommendation for provider tool kit

### Interventions

#### Reminder and recall intervention

During step 1 (current care), we will obtain key stakeholder feedback on our intervention materials and will use this feedback to optimize the overall acceptability of the materials before they are finalized. Specifically, parents of eligible children will be invited to review and provide feedback regarding the reminder-recall communication. Households of the girls and boys in the target age group of 11 to 12 years will be identified from each of the clinical practices. In randomly assigned groups of 75, parents will be invited by mailed letter to participate in individual or group interviews to evaluate the reminder-recall communication. Using the same approach as the National Immunization Survey and National Immunization Survey-Teen, the invitation will be directed toward the person in the household who is the most knowledgeable about that child’s immunizations [71]. Through individual interviews and focus groups, formative and evaluative data will be obtained on a prototype of the reminder-recall communication, to finalize the intervention for study implementation. Central features important to effective health communication will be evaluated qualitatively, including clarity, understandability, believability and convincingness, suggestions for improvement, and overall reactions.

For practices randomly assigned to the reminder-recall intervention, communication to patients due for vaccination will go out at the beginning of each calendar month. In addition, in each month, the practice will send a secure electronic communication to the parent or legal guardian of the patient through the patient’s electronic health record portal. The communication will use expectant (announcement-style or presumptive) language [33, 38]. The reminder-recall intervention will note the child’s vaccination status and state that the child’s provider strongly recommends that the child receive the HPV vaccination now. The communication also will describe the availability of topical anesthetics, indicate the availability and convenience of nurse visits for HPV vaccination, and describe the immunologic and logistic benefits of early vaccination.

This communication will encourage the parent and the patient to take advantage of the pain-reducing measures, including the nonmedication and medication approaches available at the practice. The communication will point out the availability of nurse visits that would permit vaccination without the more difficult-to-schedule provider encounter or examination. The communication will state that younger children have a higher level of antibody response to the HPV vaccine, and early vaccination takes advantage of the less busy and less complicated schedules of younger children relative to teenagers.

In this study, we will use the patient portal as the primary modality for our adolescent patients’ reminder-recall communications. We will send mailed letters to parents or guardians of patients who either have opted out of the portal messaging or do not access the portal within 1 week of delivering the reminder or recall through the portal. To support the reminder-recall intervention, we will conduct a broad education of practice staff—front desk personnel, nurses, and providers—regarding the nature of the intervention, its goals, and its likely impact on the practice.

#### Audit feedback reports and provider tool kit

The provider-facing intervention consists of two components. The first component is the missed-opportunities audit and feedback. During the steps for which the providers’ practices are randomized to this intervention, we will mail providers a monthly missed-opportunities audit and feedback report. The feedback will indicate their personal HPV vaccine missed-opportunities rate compared with the median rate of their peers and will provide the names of the top performers in their practice for the previous month. The rate will be constructed from a denominator that includes the number of visits during which they saw an 11- or 12-year-old patient eligible for a dose of HPV vaccine. The numerator will include the counts of an HPV vaccine dose ordered and given. The missed-opportunities report will undergo feedback and evaluation with a subset of providers through formative work conducted during step 1 of the protocol.

The second component will include the provision of a strong-recommendation provider tool kit. The tool kit will support providers in making strong recommendations using expectant (announcement-style or presumptive) language to express a strong recommendation [33, 38] and to address any hesitancy by engaging the parent and patient with the Corroborate, About Me, Science, Explain (CASE) approach [72, 73]. The tool kit will be an online resource available only to the staff in practices allocated to the intervention at the beginning of the step. Supervisors and research staff will communicate the availability of the tool kit through emails. To support the provider-facing intervention, we will conduct broad education of the practice staff—front desk personnel, medical secretaries, appointment secretaries, and nurses—in preparation of the intervention about the nature of the intervention, its goals, and its likely impact on the practice.

#### Process evaluation

We will assess the fidelity and quality of implementation of the interventions and evaluate at the individual, provider, and clinic levels the potential causal and contextual factors that may be associated with observed outcomes. After completion of steps 2, 3, and 4, we will survey samples of parents to evaluate their awareness of and response to the reminder-recall intervention. In addition, we will assess their attitudes and beliefs about HPV vaccination through the use of a modified version of the Carolina HPV Immunization and Attitudes and Beliefs Scale [74–76]. We will document the time to respond to the reminder-recall letter and the type of visit scheduled. Finally, we will analyze clinical records’ data to evaluate the use of pain-reducing measures and nurse-only visits. We will also survey all providers to assess their exposure to and experience with receipt of the monthly audit and feedback report and use of and experience with the strong-recommendation tool kit. We will use Web analytics to track access to the online tool kit.

After completion of steps 2, 3, and 4, we will send all providers randomly assigned to the intervention a questionnaire to assess their exposure to and experience with the audit and feedback report, use of and experience with the strong-recommendation tool kit in each step, and use of presumptive language, as well as the CASE approach in their clinical practices. Adapting items from a previously validated instrument used to survey clinicians [77], we will assess provider knowledge of HPV and HPV vaccination, perceived parental barriers to HPV vaccination, provider self-reported perceived strength of HPV vaccination recommendations, and the type of visits during which they offer the vaccine, as well as clinical practice and personal characteristics, to ascertain potential mechanisms of impact. These process measures will be used to conduct secondary analyses of the interventions’ impact on the outcomes of interest.

#### Data collection

For each 12-month period, we will electronically obtain demographic data (race/ethnicity, sex, and age) for all empaneled patients age 11 and 12 years. Our administrative database will be searched electronically to identify the occurrence and dates of all HPV vaccination of these children to the end of the study with the use of Current Procedural Terminology codes 90649, 90650, and 90651.

### Outcomes

Our primary outcome variables are the rates of at least one dose of HPV vaccine for empaneled vaccine-eligible boys and girls, measured at the end of the last day of each 12-month step. Secondary outcome variables include HPV vaccine initiation and completion rates for the eligible patients empaneled to a given practice. Specifically, we will measure at the end of the last day of each 12-month step the initiation and completion rates for the eligible patients empaneled to a given primary care practice who presented for care of any type to that practice during the 12 months of that step.

To qualify for eligibility for initiation, the patient must have been 11 or 12 years of age on the beginning day of the 12-month step. In addition, they must be due for the first dose of HPV vaccine, having never received a previous dose of the vaccine. To qualify for eligibility for completion of the HPV vaccine series, the patient must have been 11 or 12 years of age at the beginning day of the 12-month step and able to complete the series in the following 12 months, having had 0, 1, or 2 doses previously. We also will obtain rates of missed opportunities from the electronic health records. In these rates, the denominator will be the number of encounters that took place during a given step at a particular primary care practice of the empaneled patients eligible for a dose of HPV vaccine. The numerator will be the number of encounters in which an HPV vaccine was not given.

We will further categorize outcomes by patient sex, age, and vaccine dose, as well as by provider type. We will calculate the missed-opportunity rates for those providers in practices randomly assigned to receive the second intervention for aim 2 during those steps (Table 3). We also will examine the association of outcomes with variables ascertained through the process evaluation.

### Statistical analyses

We will summarize patient characteristics (sex and age) by intervention status (baseline, intervention 1, intervention 2, or interventions 1 + 2). All patients will be analyzed on an intention-to-treat status; this principle will be extended to the practice status so that delays in the implementation of an intervention will not affect the intervention status of patients [78]. We will use generalized linear mixed models to assess the effects of the interventions [78]. All models will be assessed both overall and stratified by sex in recognition of differential rates of HPV vaccination among male and female patients. Our main model will be a mixed-effects logistic regression model specified as follows.

Let *Y*_*ijt*_ indicate whether the *i*^th^ eligible patient at the *j*^th^ practice during step *t* (*t* = 0, ..., 3) receives a vaccine during period *t*. Then, as recommended by Lyons et al. [68, 69], let

$$ {Y}_{ijt}\sim \mathrm{Binomial}\left({\pi}_{ijt}\right) $$

1 $$ {\pi}_{ijt}=\mu +{a}_j+{\beta}_t+{\beta}_1X{1}_{j1}+{\beta}_2X{2}_{jt}+{\beta}_3X{1}_{jt}\times X{2}_{jt} $$

where *α*_*j*_ is a random effect for clinical practice (cluster) *j*, *j* = 1, 2, …, 6, *α*_*j*_ ~ *N*(0, *τ*^2^), β_t_ is a fixed effect for time (*t* = 0, 1, 2, 3), and *X*_*jt*_ indicates the intervention m at the *i*^th^ clinical practice at time *t*. *β*_1_ and *β*_2_ capture the main effects for the two interventions. *β*_3_ captures any interaction (synergy) effect between the two interventions. In the event of a large imbalance in any patient characteristics between baseline and intervention periods, those characteristics and potential interactions will be included in model (1). We then can test the primary aims of the study by testing *β*_1_ = 0, *β*_2_ = 0, and *β*_3_ = 0.

For secondary analyses, we will estimate models similar to the main model, using only the initiation and completion-eligible patients. For all models, we will report *C*-statistics and between-practice variance. Because of the small number of practices, estimation of between-practice variance may be computationally difficult or give biased results, or both. Thus, we will, through a sensitivity analysis, replicate the main model for all outcomes using a generalized estimating equation. The generalized estimating equation models account appropriately but conservatively for clustering of outcomes and are thus appropriate for stepped-wedge cluster randomized trials [78]. Although they have less power to detect effects, they do not require making assumptions about or estimates of the between-practice variance.

We will incorporate into our secondary analyses the measures from the process evaluation. We also will incorporate other moderating factors to determine their roles in the explanation of the magnitude and direction of the results.

#### Sample size and power

>Statistical power was estimated using simulation, which accounted for potential differences in baseline rates across sites [79]. On the basis of pilot data from a 12-month period, we have assumed an average cluster size of 800 children (400 boys and 400 girls), a baseline probability of requiring any vaccination of 25% (log odds of − 1.1), and a conservative estimate of the standard deviation of the random effect of 0.5, assuming a secular treatment effect (Table 4). These estimates are conservative. We have sufficient power to detect meaningful patient-centered and provider-level effects in both the sex-stratified and the full cohorts.

**Table 4:** Power scenarios through mixed-model simulation (no interaction)

*β* intervention	Average cluster size, no. of patients	Detectable odds ratio	Power, %
1	400	1.3	77
2	400	1.3	77
1	400	1.35	87
2	400	1.35	87
1	800	1.2	75
2	800	1.2	75
1	800	1.25	90
2	800	1.25	89

For the interaction between patient-centered and provider-level effects (with the same assumptions described above) and based on the simulation, we have at least 80% power to detect an odds ratio (OR) of 1.5 or greater when evaluating boys and girls separately and of 1.34 or greater in the full cohort (Table 5). Although our power estimates are conservative, power is somewhat marginal (70% to detect an OR of 1.45) for the synergistic effect in the sex-stratified cohorts (aim 3). However, we have sufficient power to detect an effect using the combined sample.

**Table 5:** Power scenarios through mixed-model simulation (with interaction)

*β* interventions	Average cluster size, no. of patients	Detectable odds ratio	Power, %
1	400	1.4	78
2	400	1.4	77
1 × 2	400	1.45	72
1	400	1.42	81
2	400	1.42	81
1 × 2	400	1.5	81
1	800	1.25	73
2	800	1.25	72
1 × 2	800	1.3	71
1	800	1.28	81
2	800	1.28	81
1 × 2	800	1.34	82

We conducted several sensitivity analyses with adjustment of the terms in our model (baseline probability, random effect standard deviation, and secular trend) and found small changes in our already-conservative power estimates. We used average cluster instead of actual size, which was reasonable given our pilot data indicate only a slight imbalance across the clusters [80]. Our average observed total cluster size was 895, with close to 50% male sex and 50% female sex. We assumed an average cluster size of 800 (400 for each sex) to allow for decreases in eligible participants over time as a result of the effect of our interventions.

### Data management and monitoring

For the proposed behavioral intervention, the posed risks do not exceed the threshold of minimal risk. Thus, an independent data management and monitoring committee are not required. Multiple principal investigators will monitor the safety of the participants and the integrity of the data. We will maintain patient safety through the adherence of our clinical staff to the standards of clinical care. Mayo Clinic has established uniform clinical policies and practices for all of the six practice sites, including the adoption of a standardized children and adolescent immunization schedule for southeast Minnesota and an Ambulatory Specialty Procedural Guideline for Vaccine Administration. In addition, Mayo Clinic has adopted standard nursing vaccine protocols for all routine and risk-based vaccinations. Adverse reactions to vaccinations will be managed in the clinical setting by the clinical staff as part of routine care abiding by clinic standards and federal law regarding Vaccine Adverse Events Reporting.

#### Data integrity

We will use the data integrity best practices promulgated by the Mayo Clinic Division of Biomedical Statistics and Informatics in the Department of Health Sciences Research. These include the use of real-time data validation and checklists developed and standardized for best practices.

#### Subject privacy

Institutional clinical policies and procedures maintain patient privacy with respect to communications, paperwork, and electronic health record documentation and access. Because patients will participate in the study only through their receipt of routine care, these provisions will ensure subject privacy.

#### Data confidentiality

We will ensure that all patient data are secure and confidential through adherence to institutional policies and procedures for securely storing, maintaining, and updating health record information. All health record and patient data are securely stored behind the institutional electronic firewall, and these data will not be released externally except under specific data sharing agreements. Additionally, we will store study data in separate, secure servers. Only study personnel will be able to access those servers through appropriate logins and passwords. All study results will be presented in aggregate, and no individual patient or provider will be identifiable.

#### Product accountability

All practices in this trial use a central pharmacy, which will purchase and receive all 9-valent human papillomavirus (9vHPV) vaccine. These six practices are routinely monitored by the Minnesota Department of Health to ensure adherence to the policies and procedures of vaccine storage and handling, documentation, and administration for the Minnesota Vaccines for Children Program. Mayo Clinic adheres to the Centers for Disease Control and Prevention recommendations and guidelines for vaccine storage and handling, documentation, and administration.

## Discussion

### Timeline

This is a 5-year proposal. Each design step will involve 12 months, which allows us to balance seasonality of HPV administration across the four steps [81, 82]. Step 1 involves measurement of current practice during which we will engage samples of parents and providers in optimizing the interventions. The next steps will take 36 months, leaving 12 months in year 5 for our analysis.

### Limitations and related considerations

We note potential limitations with our approach. First, the factorial design requires all steps to be completed to achieve the full power. Midtrial analyses are likely to be underpowered and at risk for type II errors. Thus, we plan no midtrial analyses. Because the study involves interventions that pose minimal risks, we do not need to conduct ongoing safety and data monitoring for this clinical trial or specify stopping rules. Second, we chose to measure the outcomes of the eligible patients’ status at each of the steps. This is opposed to following either a closed or open cohort from step to step, which may be too mathematically complex to analyze rigorously [83, 84]. Therefore, we ignore whether patients contributed to previous steps in assessing their status for a particular step. We permit patients to drop out of the provider panels, switch providers, or enter the provider panels on the basis of the primary care practices’ policies and practices regarding panel membership. Our choice simplifies eligibility at each step, as well as the mathematical analysis and results, in a conservative measure of the outcome. Third, we recognize that our interventions involve voluntary behaviors. Thus, we will use the process evaluation measures in secondary analyses to understand how intervention exposure and fidelity may impact study outcomes.

### Potential for impact and implications and plans for dissemination

The rigor, design, feasibility, and high likelihood of success of this trial will provide important evidence regarding practice and provider interventions to improve HPV vaccination. We will disseminate these findings through peer-reviewed publications, presentations, and professional meetings.

Throughout this effort, we will explore our options for sharing data in accordance with National Institutes of Health policies, with a mind toward the unique challenges around confidentiality and consent waiver of our embedded pragmatic design. Mayo Clinic is a widely dispersed organization with locations in five states. Mayo Clinic also maintains the Mayo Clinic Care Network, which consists of 45 health care organizations with a close working relationship with Mayo. Given this ready resource for dissemination, the interventions being tested in our study (if successful) can be disseminated readily to various clinical settings throughout the USA.

### Additional file

Additional file 1: The consolidated standards of reporting trials diagram for the stepped-wedge cluster randomized trial. (TIF 1392 kb)

## Appendix A

### Clinical sites participating in the trial

We will test our hypotheses in the six primary care practices at Mayo Clinic in Rochester, Minnesota, and Kasson, Minnesota, summarized in this table.

**Table 6:** Clinical sites participating in the stepped-wedge randomized trial

Clinical practice	Location
Baldwin Building Community Pediatric and Adolescent Medicine	Baldwin Building, Floor 3 221 Fourth Avenue SW Rochester, MN 55905
Baldwin Building Family Medicine	Baldwin Building, Floors 1 and 2 221 Fourth Avenue SW Rochester, MN 55905
Mayo Family Clinic Northwest	4111 West Frontage Road Hwy 52 NW Rochester, MN 55901
Mayo Family Clinic Northeast	3041 Stone Hedge Drive NE Rochester, MN 55906
Mayo Family Clinic Southeast	4544 Canal Place SE Rochester, MN 55904
Mayo Family Clinic Kasson	411 West Main Kasson, MN 55944

## Appendix B

### Description of trial setting

Mayo Clinic Employee and Community Health (ECH) serves as the practice setting for the proposed trial interventions. The ECH initiative, for which Sarah J. Crane, MD, is the practice chair, brings together Mayo Clinic’s primary care departments of Family Medicine, Primary Care Internal Medicine, and Community Pediatric and Adolescent Medicine, which include the six clinical sites (called *clusters*) identified for our trial. It embraces the Mayo Clinic Model of Community Care to provide the right care, delivered by the right provider in the right place at the right time. The goal is to deliver the highest-value care to an identified population of patients. The financial model under which ECH operates is an approach of the total cost of care. The primary focus is to deliver the highest quality and best patient care experience at the most appropriate cost to the system. To achieve this goal, ECH concentrates on core principles frequently associated with patient-centered medical homes. Care should be provided by a physician-led multidisciplinary team. All members of the team should function at the top of their licensure. Appropriate care is delivered at the right time from the right provider in the right place. Care is provided both in the office and outside of the office. ECH maintains a continuous relationship with patients through active care management. The chronic care model also serves as an important philosophy to guide practice redesign within ECH.

The six practices in our trial provide primary care for more than 151,000 empaneled patients who are cared for at seven full-service clinical practices and two express care sites. These are located in Dodge and Olmsted counties in Minnesota. There are 41 care teams of 116 physicians, including the principal investigator, Robert M. Jacobson, MD; 166 resident physicians; and 42 nurse practitioner and physician assistant providers. Patients receive reminders about necessary preventive services at the time of visits through an electronic alert system and also through mail or electronic communication on a secure patient portal that is compliant with the Health Insurance Portability and Accountability Act. Care management of chronic diseases such as asthma, diabetes mellitus, and depression is accomplished through a team approach that uses care coordinators, registered nurses, protocols, and patient education. High-use patients with multiple, often poorly controlled, chronic diseases are enrolled in a structured care management program.

In 2016, the six practices in our trial empaneled 151,322 patients, of whom 41,388 were 18 years old or younger. The 151,322 patients had a total of 985,535 unique patient encounters with 411,799 outpatient office visits from August 1, 2015, through August 1, 2016. During this time, 66,847 outpatient visits occurred for children younger than age 11 years and 32,799 outpatient visits for adolescents age 11 through 18 years. In addition, 98,112 vaccinations were given to children younger than 11 years and 49,769 vaccinations given to adolescents age 11 through 18 years. The following table shows the current distribution of 11- and 12-year-old children (the cohort of interest for this study) for sex and race/ethnicity.

**Table 7:** Distribution of 11- and 12-year-old children in the six clusters in the trial by sex and race/ethnicity

	Ethnic category, no. of patients^a^				
	Not Hispanic or Latino		Hispanic or Latino		
Race category	Female sex	Male sex	Female sex	Male sex	Total no. of patients
American Indian/Alaska Native	6	6	0	1	13
Asian	125	135	4	3	267
Native Hawaiian or other Pacific Islander	0	6	1	2	9
Black or African American	141	150	3	5	299
White	2139	2237	53	59	4488
More than one race	182	183	87	114	566
Total	2593	2717	148	184	5642

^a^The race and ethnic categories of “unknown/not reported” is not shown because, by definition, it contains no data

## Abbreviations

4vHPV: 4-Valent human papillomavirus
9vHPV: 9-Valent human papillomavirus
ACIP: Advisory Committee on Immunization Practices
CASE: Corroborate, About Me, Science, Explain
HPV: Human papillomavirus
IRB: Institutional review board
OR: Odds ratio
SEM: Social ecological model
