# Supplementary figures and images for: Evaluating the impact of multilevel evidence-based implementation strategies to enhance provider recommendation on human papillomavirus vaccination rates among an empaneled primary care patient population: a study protocol for a stepped-wedge cluster randomized trial
Source: Implement Sci. 2018 Jul 13;13:96. doi: 10.1186/s13012-018-0778-x (PMC6043954; doi:10.1186/s13012-018-0778-x)

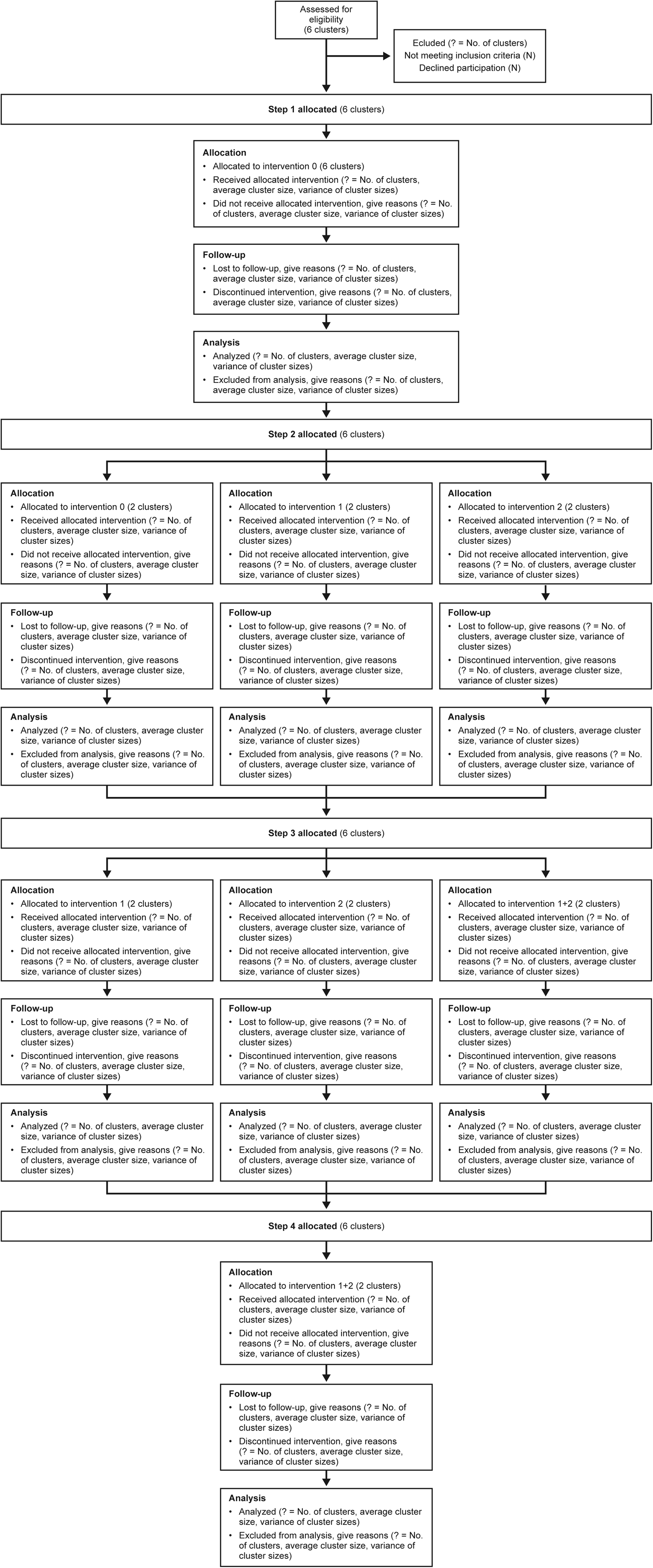

Supplement: Supplementary file 1 — The consolidated standards of reporting trials diagram for the stepped-wedge cluster randomized trial. (TIF 1392 kb) [file 13012_2018_778_MOESM1_ESM.tif]
